# Depression and Deliberate Self-Harm Among Rural Adolescents of Sichuan Province in Western China: A 2-Year Longitudinal Study

**DOI:** 10.3389/fpsyt.2021.605785

**Published:** 2021-09-13

**Authors:** Shimin Lai, Chang Su, Shasha Song, Mingxia Yan, Chengmeng Tang, Qiang Zhang, Fei Yin, Qiaolan Liu

**Affiliations:** ^1^Department of Health Behavior and Social Medicine, West China School of Public Health and West China Fourth Hospital, Research Center for Palliative Care, West China-PUMC C.C. Chen Institute of Health, Sichuan University, Chengdu, China; ^2^Department of Medical Administration, West China Tianfu Hospital, Sichuan University, Chengdu, China; ^3^Department of Epidemiology and Health Statistics, West China School of Public Health and West China Fourth Hospital, Sichuan University, Chengdu, China

**Keywords:** depression, deliberate self-harm, rural adolescents, repeated measures, bivariate multilevel logistic regression

## Abstract

**Objective:** To explore the change in the prevalence and association of depression and deliberate self-harm and their common and independent influencing factors among western Chinese rural adolescents.

**Methods:** A total of 2,744 junior and senior high school students from two rural schools in Sichuan Province, China, participated in the baseline survey and were invited to participate in two follow-up surveys. The Center for Epidemiologic Studies-Depression Scale, a deliberate self-harm item, the Social Support Rating Scale, the Rosenberg Self-esteem Scale and the Connor-Davidson Resilience Scale were administered. A bivariate four-level logistic regression model was used for analysis.

**Results:** The prevalence of depression and deliberate self-harm were 39.6 and 21.2%, respectively. Regular physical exercise, a good relationship with parents, high resilience, and high self-esteem were common protective factors for both depression and deliberate self-harm. Feeling disliked by teachers was a common risk factor for both. Being female, having a mother who emigrated as a migrant worker before the student was 3 years old, feeling disliked by classmates and having a poor family economic status were associated only with an increased risk of depression. Participants with medium social support were less likely to report deliberate self-harm than those with low or high support. Depression and deliberate self-harm were clustered at the class level.

**Conclusions:** The comorbidity of depression and deliberate self-harm in rural adolescents should be given ample attention. Interventions should consider the class clustering of depression and deliberate self-harm and their common and unique influencing factors.

## Introduction

Depression and deliberate self-harm (DSH) in adolescents are two public health issues that deserve serious attention. Depression is a common mental disorder during adolescence. Previous studies have shown that depression mostly first appears during adolescence (1, 2). Many adolescents experience depression at least once during adolescence and may experience repeated episodes (3). Depression is widely considered to be a significant predictor of suicide and may predict adverse outcomes such as bipolar disorder and anxiety in adulthood (2, 4). Previous studies have reported that physical inactivity, school bullying, and prolonged parental separation can all contribute to depression in adolescents and that girls generally have higher rates of depression than boys (1, 5–7). Therefore, the implementation of early screening and intervention for depression in adolescents is an important public health issue.

According to previous research, DSH usually refers to behavior that directly or intentionally causes damage to one's own body or mind, regardless of the type of motivation or the level of suicidal intent (8). DSH usually has the following functions: emotional regulation, anti-dissociation, anti-suicide, interpersonal boundaries, interpersonal-influence, self-punishment, and sensation-seeking. Among them, emotional regulation mainly relieves painful emotions, interpersonal-influence refers to seeking help from others, self-punishment refers to expressing anger toward oneself, and sensation-seeking refers to seeking stimulation (9). DSH often involves repeated harm. Adolescents who engage in DSH have a series of negative consequences, such as mental disorders and substance abuse, in their early adult years and, more importantly, an increased risk of suicide later in life (10, 11). According to the Multicenter Study of DSH in England, which followed people who had engaged in DSH for 10 years, the potential risk of suicide rose to 0.6% (1.01% for men and 0.31% for women) and 57 times the risk of the general population (53 times for men and 68 times for women) in the first year after DSH (12). Therefore, DSH prevention should be the first line of defense to reduce the risk of adolescent suicide. The contributing factors of DSH include common negative daily life events, such as poor relationships with parents, early adversity and poor social interpersonal relationships with peers and teachers (13, 14). There may also be sex differences in DSH, with one community study showing that girls were four times more likely to engage in DSH than boys (15).

A positive association between depression and DSH has been widely reported. DSH is a kind of emotion regulation behavior (16, 17). A previous Chinese study showed that an increase in depression among teenagers independently increases the risk of DSH (18). Adolescents who engage in DSH behavior have an increased risk of developing depression in adulthood (5). These studies suggest that depression and DSH may be concomitant in the adolescent population. Whether the same or different factors affect depression and DSH is worth exploring.

Chinese rural adolescents may face more mental health problems due to the influence of the family environment and social environment (6). Among the influencing factors of adolescent depression and DSH, psychological factors such as social support, resilience, and self-esteem have been widely considered (19). Studies have found that social support, resilience and self-esteem affect adolescent depression and DSH, and this influence is usually a protective measure. However, after considering depression positively associated with DSH and presenting simultaneously in the same adolescent individuals, whether these psychological variables have a similar protective effect in rural adolescents is unclear.

In the social and economic development of China, the western region has lagged behind the eastern region, and rural areas have lagged behind urban areas. There are few health resources for children and adolescents in western Chinese rural areas. In addition, many young parents migrate to cities to work, reducing the amount of time they spend with their children, which possibly increases psychological and behavioral problems in children and adolescents (6). Due to a lack of effective guardians in the family, depression and DSH among adolescents in these areas have received insufficient attention. Adverse experiences in childhood not only increase DSH by directly affecting personality formation and increasing negative emotions but also by acting on various biological substances through genes. Childhood neglect/interruption of parental attachment may lead to increased mood disorders and DSH by reducing oxytocin (6, 20).

In this study, based on repeated measurement data from a 2-year longitudinal study in western Chinese rural adolescents and a bivariate logistic regression model, we used both depression and DSH as dependent variables to achieve the following aims:

1. Understand the current situation of depression and DSH among rural adolescents in Sichuan Province, China.
2. Explore the common factors affecting both depression and DSH and the independent factors affecting depression and DSH.
3. Examine whether the prevalence of depression and DSH is clustered at the class level.

## Materials and Methods

### Participants

A 2-year (October 2015–2017) longitudinal study was conducted in two schools (including junior high schools for grades 7–9 and senior high schools for grades 10–12) in Sichuan Province, China. Due to the accessibility of the research subjects and feasibility of the study procedures, typical sampling and cluster sampling methods were adopted. In the first stage, typical sampling was performed. Zizhong County was selected because it represents the average level of economic and social development in western rural areas. In the second stage, we randomly selected two schools from 17 rural high schools in Zizhong County. Grade 7 students from the junior high school and grade 10 students from the senior high school of these two schools, totaling 42 classes, were included as research subjects.

The baseline survey was conducted in October 2015, and then four follow-up surveys were performed in the same adolescent population every 6 months until October 2017. Both depression and DSH were investigated in the baseline survey, the second follow-up survey and the fourth follow-up survey; therefore, in this study, we included these data from these three time points for analysis.

The effective sample sizes for the three surveys were 2,869, 2,750, and 2,457. The data from the second follow-up survey and fourth follow-up survey were matched with the baseline data, and the participants were excluded if they did not complete the depression scale or the DSH item. Then, the remaining data were included for analysis regardless of whether the subjects participated in the investigation three times, two times or just one time. Finally, 2,744 rural adolescents were included (2,744 participants in the baseline survey, 2,320 participants in the second follow-up survey, and 1,950 participants in the fourth follow-up survey). The differences in the sex ratio and grade ratio between the participants in the three surveys whom we excluded and the participants whose data we analyzed in this study were not significant. In the baseline survey, there were 1,308 boys (47.7%), 1,436 girls (52.3%), 554 junior high school students (20.2%) and 2,190 senior high school students (79.8%). On average, the participants were 14.7 years old (SD: 1.4; Range: 10–19). All subjects gave their informed consent for inclusion before they participated in the study. Informed consent was obtained from the parents and guardians. The ethical approval of the data collection was obtained from the Medical Ethical Committee of Sichuan University (No. 20140307).

### Instruments

#### Sociodemographic Characteristics

Demographic data were collected by a self-report questionnaire with questions on students' sex, age, grade, family economic status, age when their father/mother emigrated as a migrant worker, feeling about being liked or disliked by teachers/classmates, relationship with parents, family structure, parents' education level, and time spent with parents every year.

#### Deliberate Self-Harm

The study used the following question to assess DSH: “How many times have you engaged in deliberate self-harm behavior (e.g., deliberately cutting, burning, scratching, hitting body parts, or biting yourself) in the past 12 months?” The response options included ① none, ② once, ③ 2~3 times, and ④ ≥4 times; answers from ② ~ ④ were recorded as deliberate self-harm. Before the formal survey, we selected 50 middle school and high school students to conduct a preliminary survey. A week later, we conducted a repeat survey, and the rank correlation coefficient of the DSH from the two surveys was 0.81. Further, we conducted rank correlation analysis on the 3-time original data by DSH score (1–4) from the baseline, the second follow-up and the fourth follow-up survey. The results showed that the rank correlation coefficient of the first data with the second data was 0.197, the rank correlation coefficient of the first data with the third data was 0.219, and the rank correlation coefficient of the second data and the third data was 0.311. Both *P*-values were <0.05, showing statistical significance (Appendix Table 1).

#### Depression

The Center for Epidemiologic Studies-Depression Scale (CES-D), a self-rating depression scale that assesses depression and psychological conditions, is often used for the comparison of survey results over time (21). The CES-D has 20 items. A score <16 indicates no depression, while a score of 16 or more indicates depression (22). In this study, Cronbach's α was 0.943.

#### Self-Esteem

The Rosenberg Self-esteem Scale (SES) developed by Rosenberg is composed of 10 items, all of which are rated on a Likert-type scale ranging from 1 (strongly disagree) to 4 (strongly agree). The sum of the item scores is the total score (23). The higher the total score is, the higher the individual's self-esteem level. The cutoff points selected for this study (the 27th and 73rd percentiles) are the recommended values for item discrimination when the purpose is to work with “high” and “low” groups in any dimension from a statistical point of view (24, 25). The SES has shown good reliability and validity among Chinese children and adolescents (26). In the current study, Cronbach's α was 0.809.

#### Resilience

The Connor-Davidson Resilience Scale consists of 25 items and was developed by Connor and Davidson in 2003 (27), consists of 25 items and is the most commonly used scale to measure individual mental resilience in academic circles at home and abroad. The 25 items are evaluated on a five-point scoring scale. The higher the total score, the better the mental resilience. The cutoff points selected for this study (the 27th and 73rd percentiles) are the recommended values for item discrimination and are the same as those selected for the SES. In this study, Cronbach's α was 0.900.

#### Social Support

Social support was measured by the Chinese version of the Social Support Rating scale (SSRS), which was developed by Xiao S.Y. (28). The total social support score is the sum of the scores of all items, with a higher score reflecting higher social support. The cutoff points selected for this study (the 27th and 73rd percentiles) are the recommended values for item discrimination and are the same as those selected for the SES. In the current study, Cronbach's α was 0.779.

### Data Analysis

**Figure 1:**
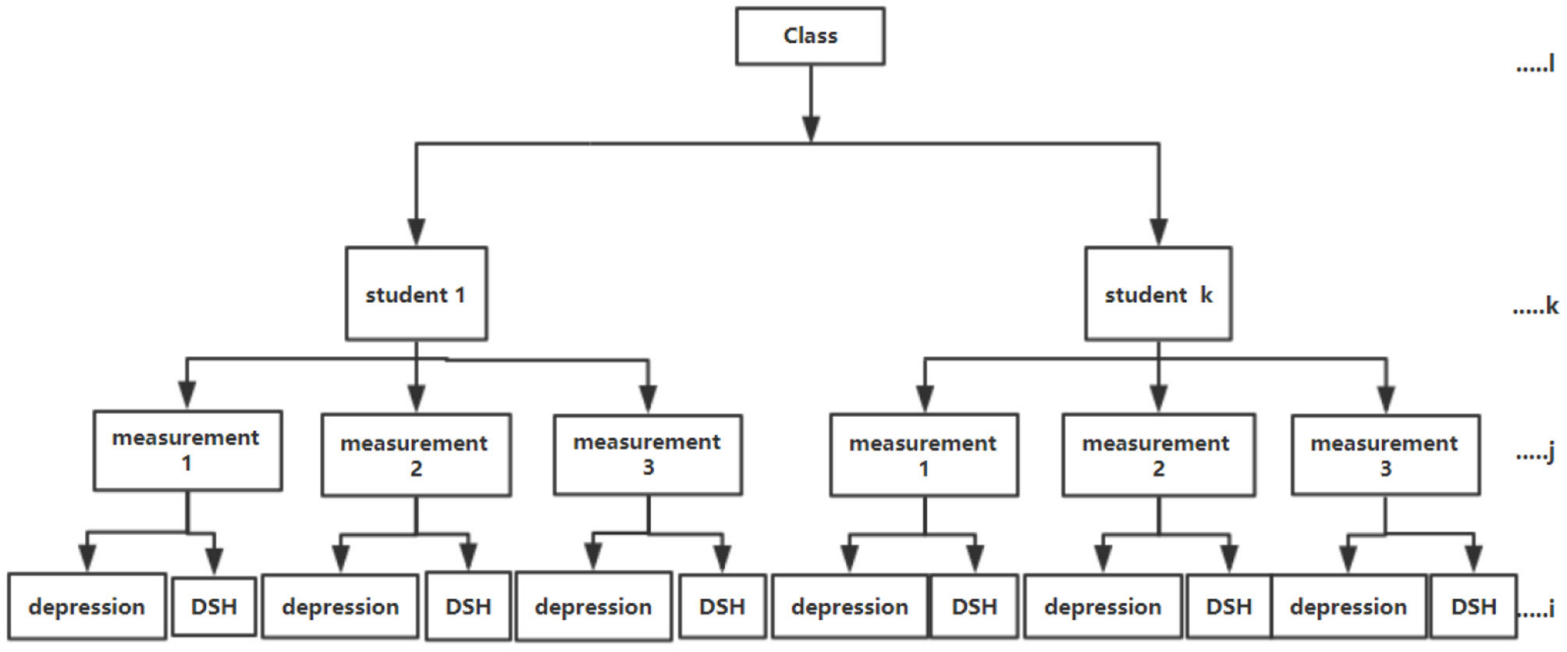
The hierarchical structure of depression and deliberate self-harm (DSH) in rural adolescents.

This study used SPSS 22.0 (IBM; Armonk, New York, NY) for data processing and statistical analysis. Based on repeated measurement data, MLwin 2.02 was used to establish a bivariate four-level logistic regression model of depression and DSH and their influencing factors. We first estimated the binary association between depression and DSH, with a correlation of 0.217, indicating an association between the two outcomes. The hierarchical structure of the data in this study is shown in Figure 1. We constructed a zero model without any explanatory variables using formula (1). The purpose of this model was to understand whether there was variation in the two response variables when the explanatory variables were not included. π_1jkl_ represents the prevalence of depression, and π_2jkl_ represents the prevalence of DSH (29).


(1) $$\left\{ \begin{array}{l} logitP_{1}=ln\left(\pi_{1jkl}/\left(1-\pi_{2jkl}\right)\right)=\left(\beta_{2}+\nu_{2kl}+f_{2l}\right)Z_{1jkl}\\ logitP_{2}=ln\left(\pi_{2jkl}/\left(1-\pi_{1jkl}\right)\right)=\left(\beta_{1}+\nu_{1kl}+f_{1l}\right)Z_{2jkl} \end{array}\right.$$

$$\begin{array}{l} Z_{1jkl}\left\{ \begin{array}{l} 1depression\\ 0deliberateselfharm \end{array}\right.Z_{2jkl}\left\{ \begin{array}{l} 0depression\\ 1deliberateselfharm \end{array}\right.\\ \;\left[\begin{array}{l} f_{1l}\\ f_{2l} \end{array}\right]\sim N(0,\Omega_{f}),\;\Omega_{f}=\left[\begin{array}{l} \sigma_{f_{1}}^{2}\\ \sigma_{f_{12}}\sigma_{f_{2}}^{2} \end{array}\right],\\ \;\left[\begin{array}{l} \nu_{1kl}\\ \nu_{2kl} \end{array}\right]\sim N(0,\Omega_{\nu }),\;\Omega_{\nu }=\left[\begin{array}{l} \sigma_{\nu_{1}}^{2}\\ \sigma \nu_{12}\sigma_{\nu_{2}}^{2} \end{array}\right] \end{array}$$

β_1_ and β_2_ are the intercept items, ν_1kl_ and ν_2kl_ represent the variation among students, and *f*_1l_ and *f*_2l_ represent the variation among classes. *Z*_1*kjl*_ and *Z*_2*kjl*_ are the indicated variables. The explanatory variables were added to the model if the variances of high levels had statistical significance. The independent explanatory variables or common explanatory variables were included in the model according to the generalized Wald test.

## Results

The total prevalence of depression in rural adolescents in Sichuan Province was 39.6%. At baseline, the prevalence was 34.5%; at the second follow-up survey, it was 46.3%; and at the fourth follow-up survey, it was 39.0%. The changes in depression and DSH in junior high school and senior high school students over 3 years are shown in Table 1.

**Table 1:** The changes in depression and DSH in junior high school and senior high school students.

		**Depression**		**DSH**	
		**No (%)**	**Yes (%)**	**No (%)**	**Yes (%)**
Junior high	7	393 (70.9)	161 (29.1)	452 (81.6)	102 (18.4)
school	8	252 (51.4)	238 (48.6)	262 (53.5)	23 (48.6)
	9	202 (51.5)	190 (48.5)	328 (83.7)	64 (16.3)
Senior high	10	140 (64.2)	783 (35.8)	1,800 (82.3)	386 (17.7)
school	11	993 (63.4)	837 (45.7)	1,260 (68.9)	570 (31.1)
	12	989 (63.4)	571 (36.6)	1,423 (91.2)	137 (8.8)

Table 2 shows the demographic characteristics of the 2,744 students. The results of the univariate test showed that the common factors affecting the prevalence of depression and DSH in students included sex, feelings about being liked or disliked by teachers and classmates, relationship with parents, family economic status, physical exercise, resilience, social support, and self-esteem (*P* < 0.001). The independent factors affecting depression were age and age when one's father or mother emigrated as a migrant worker, while the independent factor of DSH was family structure.

**Table 2:** Single factor test of depression and DSH.

**Variable**	**Depression**		**χ^2^**	**DSH**		**χ^2^**
	**No (%)**	**Yes (%)**		**No (%)**	**Yes (%)**	
**Sex**						
Male	905 (69.6%)	396 (30.4%)	18.37^***^	1094 (84.1%)	207 (15.9%)	6.36^*^
Female	885 (61.8%)	548 (38.2%)		1,152 (80.4%)	281 (19.6%)	
**Age**						
<15 years old	528 (69.3%)	234 (30.7%)	6.55^*^	452 (81.6%)	102 (18.4%)	0.17
≥15 years old	1,268 (64.1%)	710 (35.9%)		1,800 (82.3%)	386 (17.7%)	
**Family structure**						
Intact	1,565 (65.9%)	809 (34.1%)	1.76	1,967 (82.9%)	407 (17.1%)	5.75^*^
Divorce/death of one or both parent	220 (62.3%)	133 (37.7%)		274 (77.6%)	79 (22.4%)	
**Left-behind adolescent**						
No	796 (67.3%)	387 (32.7%)	2.77	984 (83.2%)	199 (16.8%)	1.20
Yes	982 (64.2%)	547 (35.8%)		1,247 (81.6%)	282 (18.4%)	
**Father's education**						
Primary School or Below	465 (63.4%)	268 (36.6%)	4.54	587 (80.1%)	146 (19.9%)	3.79
Junior-senior high school	1,155 (65.8%)	601 (34.2%)		1,456 (82.9%)	300 (17.1%)	
College degree and above	69 (74.2%)	24 (25.8%)		80 (86.0%)	13 (14.0%)	
**Mother's education**						
Primary School or Below	570 (63.1%)	334 (36.9%)	4.65	729 (80.6%)	175 (19.4%)	3.99
Junior-senior high school	1,039 (66.6%)	522 (33.4%)		1,283 (82.2%)	278 (17.8%)	
College degree and above	50 (72.5%)	19 (27.5%)		62 (89.9%)	7 (10.1%)	
**Age when one's father emigrated as a migrant worker**						
≤ 3 years old	979 (63.1%)	573 (36.9%)	8.95^**^	1,275 (82.2%)	277 (17.8%)	0.07
>3 years old	775 (68.6)	354 (31.4%)		932 (82.6%)	197 (17.4%)	
**Age when one's mother emigrated as a migrant worker**						
≤ 3 years old	730 (69.6%)	319 (30.4%)	12.34^***^	861 (81.2%)	188 (17.9%)	0.001
>3 years old	1026 (63.0%)	603 (37.0%)		1,338 (82.1%)	291 (17.9%)	
**Feeling disliked by teachers**						
Like	1,384 (72.7%)	519 (27.3%)	158.34^***^	1,625 (85.4%)	278 (14.6%)	50.71^***^
Dislike	372 (47.3%)	414 (52.7%)		580 (73.8%)	206 (26.2%)	
**Feeling disliked by classmates**						
Like	1,561 (71.7%)	615 (28.3%)	197.32^***^	1,835 (84.3%)	341 (15.7%)	40.82^***^
Dislike	204 (39.2%)	317 (60.8%)		377 (72.4%)	144 (27.6%)	
**Relationship with parents**						
Poor	87 (47.8%)	95 (52.2%)	66.53^***^	1,768 (85.1%)	310 (14.9%)	50.26^***^
Medium	246 (54.1%)	209 (45.9%)		340 (74.7%)	115 (25.3%)	
Good	1,445 (69.5%)	633 (30.5%)		126 (69.2%)	56 (30.8%)	
**Family economic status**						
Poor	370 (58.9%)	258 (41.1%)	15.24^***^	64 (79.0%)	17 (21.0%)	10.50^**^
Medium	1,333 (67.4%)	644 (32.6%)		1,652 (83.6%)	325 (16.4%)	
Rich	53 (65.4%)	28 (34.6%)		490 (78.0%)	138 (22.0%)	
**Lack of exercise**						
Yes	1,002 (62.1%)	611 (37.9%)	19.13^***^	1,299 (80.5%)	314 (19.5%)	6.70^**^
No	745 (70.3%)	314 (29.7%)		895 (84.5%)	164 (15.5%)	
**Resilience**						
Low	390 (50.3%)	385 (49.7%)	123.80^***^	600 (77.4%)	175 (22.6%)	14.97^**^
Medium	870 (70.4%)	366 (29.6%)		1,031 (83.4%)	205 (16.6%)	
High	427 (76.8%)	129 (23.2%)		470 (84.5%)	86 (15.5%)	
**Self-esteem**						
Low	422 (51.4%)	399 (48.6%)	150.82^***^	624 (76.0%)	197 (24.0%)	43.31^***^
Medium	793 (70.9%)	325 (29.1%)		949 (84.9%)	169 (15.1%)	
High	437 (81.8%)	97 (18.2%)		474 (88.8%)	60 (11.2%)	
**Social support**						
Low	329 (48.5%)	349 (51.5%)	155.79^***^	504 (74.3%)	174 (25.7%)	48.08^***^
Medium	927 (66.5%)	466 (33.5%)		1155 (82.9%)	238 (17.1%)	
High	540 (80.7%)	129 (19.3%)		593 (88.6%)	76 (11.4%)	

*All statistical tests were two-tailed*.

* *Significance at the 0.05 level (2-tailed)*.

** *Significance at the 0.01 level (2-tailed)*.

*** *Significance at the 0.001 level (2-tailed)*.

The four-level zero model was fit without any explanatory variables (Model 1). The level 4 variance of the model was statistically significant ($\chi_{\text{wald}}^{2}$ = 27.33, *df* = 3, *P* < 0.001). Combined with professional knowledge, the data were aggregated at a high level, and the hierarchical structure of the model could not be ignored.

**Figure 2:**
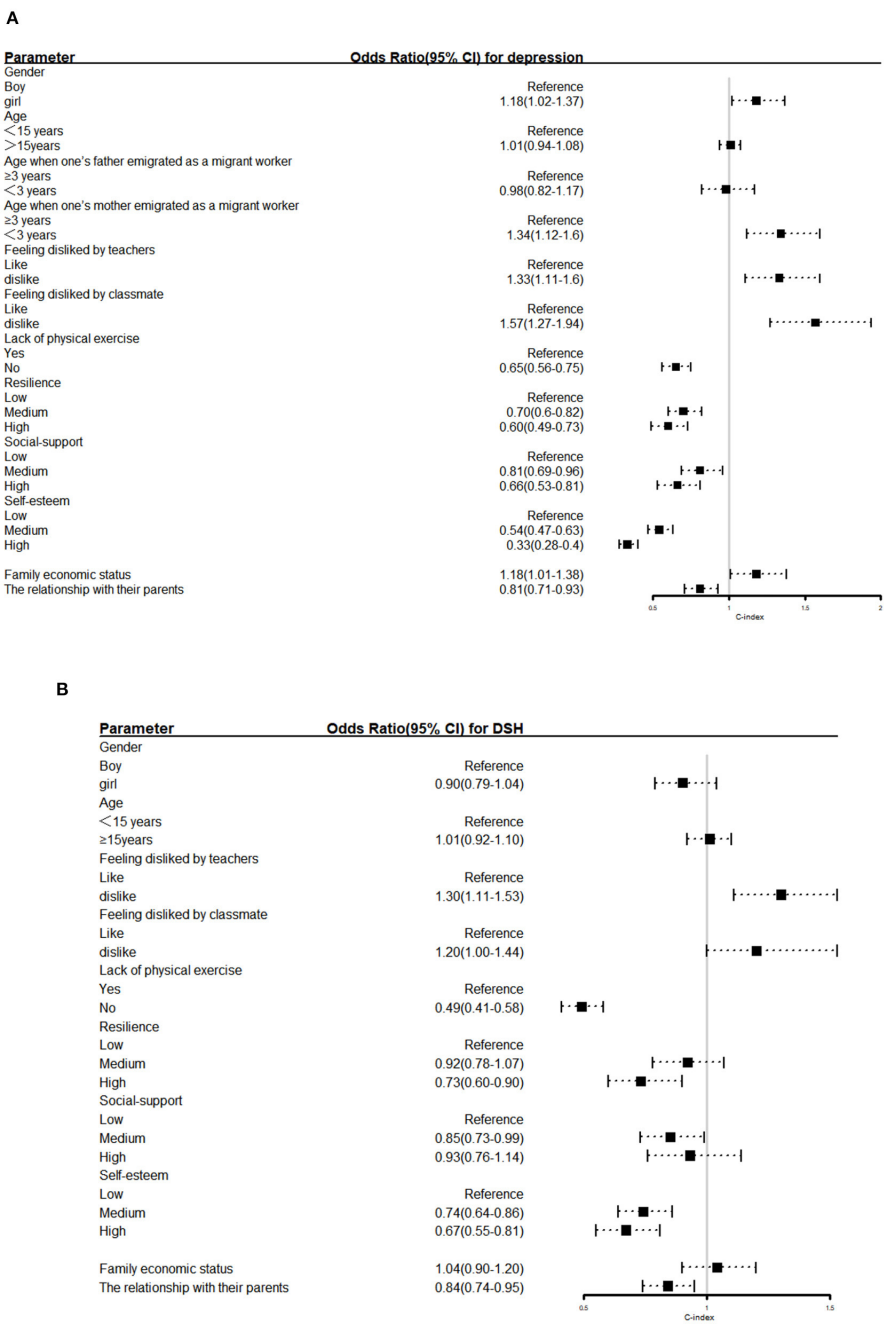
Odds ratios for **(A)** depression and **(B)** deliberate self-harm(DSH). The horizontal lines represent the 95% confidence interval (CI).

Then, the explanatory variables were also included in the model (Model 2). Better relationships with parents, regular physical exercise and high/medium self-esteem were the common protective factors for both depression and DSH. Feeling disliked by teachers was a common risk factor for both depression and DSH. Independent factors that increased only the risk of depression included being female, having a mother who emigrated as a migrant worker before the student was 3 years old, feeling disliked by classmates, and having a poor family economic status. Adolescents with high and medium resilience were less likely to have depression. Those with high and medium social support had a lower risk of depression. Those with medium social support and high resilience were less likely to report DSH. The odds ratio is shown in Figure 2. In addition, the random effect results showed that the three- and four-level variances were statistically significant, suggesting clustering at the class level and individual level. The covariance and correlation coefficients at the three levels were statistically significant, indicating a correlation of depression and DSH in the three repeated measurements (Table 3).

**Table 3:** Bivariate four-level logistic regression model of depression and DSH.

**Effect**	**Parameter**	**Model 1**		**Model 2**	
		**Depression**	**DSH**	**Depression**	**DSH**
		**Coeff. (SE)**	**Coeff. (SE)**	**Coeff. (SE)**	**Coeff. (SE)**
**Fixed effect**					
	β_0_	−0.546 (0.053)^*^	−1.537 (0.082)^*^	0.134 (0.608)^*^	−0.386 (0.769)^*^
	Sex (Boy)				
	Girl			0.168 (0.074)^*^	−0.101 (0.070)
	Age (<15 years)				
	≥15 years			0.009 (0.035)	0.009 (0.046)
	Family structure (Intact)				
	Divorce/death of one or both parents				−0.004 (0.105)
	Feeling disliked by teachers (Like)				
	Dislike			0.287 (0.092)^*^	0.264 (0.083)^*^
	Feeling disliked by classmates (Like)				
	Dislike			0.450 (0.108)^*^	0.181 (0.095)
	The relationship with their parents			−0.207 (0.067)^*^	−0.177 (0.063)^*^
	Family economic status			0.167 (0.078)^*^	0.036 (0.073)
	Lack of physical exercise (Yes)				
	No			−0.430 (0.074)^*^	−0.723 (0.088)^*^
	Age when one's father emigrated as a migrant worker (≥3 years)				
	<3 years			−0.021 (0.089)	–
	Age when one's mother emigrated as a migrant worker (≥3 years)				
	<3 years			0.294 (0.090)^*^	–
	Resilience (Low)				
	Medium			−0.361 (0.080)^*^	−0.088 (0.079)
	High			−0.512 (0.100)^*^	−0.309 (0.103)^*^
	Social-support (Low)				
	Medium			−0.205 (0.086)^*^	−0.161 (0.078)^*^
	High			−0.422 (0.110)^*^	−0.074 (0.103)
	Self-esteem (Low)				
	Medium			−0.611 (0.078)^*^	−0.299 (0.078)^*^
	High			−1.096 (0.095)^*^	−0.402 (0.096)^*^
**Random effect**					
Level 4 (class)	$\sigma_{\text{f}}^{2}$	0.067 (0.036)	0.501 (0.099)^*^	0.051 (0.024)^*^	0.473 (0.094)^*^
	$\sigma_{{\text{f}}_{\text{12}}}^{}$	0.102 (0.046)^*^	0.108 (0.038)^*^
Level 3 (individual)	$\sigma_{\nu }^{2}$	3.002 (0.146)^*^	0.972 (0.094)^*^	1.433 (0.082)^*^	0.355 (0.079)^*^
	$\sigma_{\nu_{12}}^{}$	0.922 (0.089)^*^	0.217 (0.059)^*^
Level 2 (repeated measurement)	ρ	0.113 (0.012)^*^	0.112 (0.014)^*^
Level 1 (dummy variable)	δ	0.757 (0.016)^*^	0.758 (0.016)^*^	0.811 (0.019)^*^	0.860 (0.019)^*^

–*There were no statistically significant entries after a single factor analysis*.

*–Relationship with parents: 0 Poor, 1 Medium, 2 Good; Family economic status: 0 Good, 1 Medium, 2 Poor*.

* *Significance at the 0.05 level (2-tailed)*.

*-ρ: Covariance or correlation coefficient*.

*–In the fixed effects section of explanatory variables, the categories in brackets are reference groups*.

## Discussion

Based on a 2-year longitudinal design, the overall prevalence of depression and DSH among children and adolescents in rural areas of Sichuan Province, China, were 39.6 and 21.2%, respectively. Better relationships with parents, regular physical exercise and high self-esteem were the common protective factors for both depression and DSH in adolescents. Feeling disliked by teachers was a common risk factor for both depression and DSH. Independent factors that increased only the risk of depression included being female, having a mother who emigrated as a migrant worker before the student was 3 years old, feeling disliked by classmates, and having a poor family economic status. High and medium resilience were associated with a lower risk of depression than low resilience. High and medium social supports were associated with a lower risk of depression than low social support. Medium social support and high resilience were associated with a lower risk of DSH than low social support and low resilience. Moreover, depression and DSH were clustered in the same classes.

Mental health problems and risky health behaviors among rural children in economically underdeveloped areas are worrisome. In previous studies using the same scale, the prevalence of depression in Chinese adolescents was ~23–32% (30, 31), but our results were much higher. The rate of DSH in adolescents in past studies was reported to be 5–37% (32), and adolescents in this study had a higher prevalence of DSH than those in developed areas, such as Guangdong Province in China (6.2%) (33) and Europe (5.6%)(15).

The grade distribution of depression and DSH showed some regularity. For junior high school students, the prevalence of depression increased significantly after grade 7 and reached a higher level in grade 8 and grade 9, indicating that grade 8 is an important turning point in the prevalence of depression. In this study, 88.8% of the students in grade 8 and grade 9 were aged 12–15 years old, had entered puberty and were in a period of drastic physical and mental changes (34). In this period, adolescents face hormonal changes during puberty, an increase in negative life events, and interpersonal challenges (35). Our results are consistent with previous studies, in which depression usually began in early adolescence (ages 10–14) and reached its peak in mid-adolescence (ages 14–17) (36). Moreover, in China, the high school entrance examination is regarded as the most important event in the 9 years of compulsory education. Teachers and parents attach great importance to students' studies, and some schools even advance to the grade 9 curriculum in grade 8. In addition, at the end of grade 8, junior high school students complete exams for certain subjects, which causes great academic pressure. All of these are possible causes of depression. The change in the prevalence of depression in senior high school was similar to that in junior high school, reaching a peak in grade 11 but showing a slight decrease in grade 12, the last year before the National College Entrance Examination. For ordinary Chinese students, the National College Entrance Examination may determine the course of their lives. Teachers and parents understand the pressure on adolescents, so they may be more caring and exert less pressure at this time, leading to a decrease in both depression and DSH in grade 12. Previous studies have also shown that depression begins to decrease during the transition from late adolescence (after age 17) to early adulthood. Notably, in the period from grades 7–12, the prevalence of DSH was the highest in grade 8. In previous studies, 12 years old was generally considered to be the age at which children are at the highest risk for DSH (37). Another study also showed that the average age when junior high school students in Wuhan, China engaged in DSH was 12.4 years old (38). In the current study, the average age of eighth graders who engaged in DSH was 13.2, slightly older than that in the Wuhan study.

This study found that depression and DSH were concomitant, which was consistent with previous studies (16). From the perspective of biological factors, 5-HT2, an important neurotransmitter of emotion regulation, is very important for maintaining emotional stability. Low levels of 5-HT2 in the cerebral cortex can cause negative emotions such as depression, and the prevalence of DSH will also increase (39). Further research on the biological mechanisms underlying the relationships revealed in our study is needed. In addition to the neurobiological mechanisms, research shows that emotional regulation in depression associated with DSH plays a strong role in mediation; depressed individuals often use more negative emotion regulation strategies. However, some scholars have identified that DSH is essentially an effective affective disorder caused by the maladjustment of coping strategies; therefore, depression and DSH usually have a significant correlation (40).

Having a good relationship with parents reduced the risk of depression and DSH in adolescents. According to attachment theory, adult-child relationships can have an impact on the personality development and mental health of adolescents. In addition, as adolescents grow older, parent-child conflicts usually increase, leading to parent-child tension (41). Without correct guidance or psychological counseling, adolescents' negative emotions increase, and extreme behaviors can occur. Therefore, a good parent-child relationship is of great importance to the physical and mental development of adolescents.

Taking an active part in physical exercise could reduce depression and DSH in adolescents. Previous studies have shown that regular participation in physical exercise can not only enhance adolescents' confidence but also help adolescents develop social relationships through their participation in cooperative activities such as basketball and football, thus having a positive impact on mental health (7, 42).

A higher level of self-esteem was a protective predictor of depression and DSH, consistent with previous studies (43, 44). The model of interpersonal relationships, self-esteem, and depression susceptibility proposed by Orth et al. shows that individuals with low levels of self-esteem are more inclined to engage in social avoidance and have less social support, which will increase their risk of depression (45). Another characteristic of individuals with low levels of self-esteem is that they are prone to self-blame, which is one of the characteristics of DSH. In previous studies, When adolescents have self-blame or pessimistic attribution style, they may be more likely to use self-punishment as a self-management technique. DSH is one of the techniques of self-punishment, so DSH is a strategy to relieve the tension and pain caused by self-blame (46). A brief but targeted assessment of self-esteem, focusing on factors closely associated with DSH, may help identify adolescents at risk for DSH in the future (47).

Consistent with previous research, both the parent-child relationship and the teacher-student relationship influenced the mood and behavior of adolescents (48, 49). The two schools in our study were boarding schools, so adolescents spent much time with teachers, and the teacher-child relationship was important. Teachers played an important role in the growth of these adolescents. If students could not feel the trust of teachers and have a good relationship with teachers in the teaching process, emotional and behavioral problems could ensue.

Having a mother who emigrated to work before the student was 3 years old increased only depression during adolescence. In China, children usually start kindergarten after the age of three. Children separated from their parents or mothers at a younger age experience impacts on their language learning, early habits, and emotional support. A child's mother is usually his or her earliest and most important attachment subject. In terms of children's learning, nurturing and emotional companionship, the role of the mother is often more important than that of the father (50), so the absence of early maternal companionship leads to increased depression later in the child's life (51). Therefore, it is highly recommended that mothers try to be with their children until the age of three. After leaving their children, mothers should also talk to them by phone or video regularly and make time to come home to visit them often.

Medium social support could reduce the prevalence of DSH, but higher social support did not reduce the likelihood of DSH. Previous studies have shown that when an individual's DSH is discovered, perceived support increases, but this perceived support leads to a stronger impulse to engage in DSH (52). According to the theory of psychological needs, Suyemoto suggested that some adolescents receive more attention and care from the outside world after engaging in DSH, and when they lack such attention, they will repeat DSH behavior, thus reinforcing this behavior. In other words, DSH is a means for adolescents to seek social attention and support. If they receive too much attention after DSH, they may experience repeated DSH behaviors (53), so high social support may not reduce DSH. A moderate level of social support should be provided to these adolescents. Too little or too much is not appropriate.

Adolescents in the same class had similar risks of depression and DSH. In China, students basically stay in the same class throughout their years of school before they enter a university. Adolescents spend most of their time interacting with their regular classmates, so peer contagion can occur among students in the same class. Peer contagion is a process of mutual influence between individuals and peers, which may occur in relation to negative behaviors and emotions, including aggression, bullying, poor diet and depression (54), and the influence reaches its peak in adolescence (55). Classmates are likely to interact with each other in class, and therefore, if there are adolescents with depression and DSH behaviors in the class, a higher prevalence of depression and DSH can result in some classes. In addition, the teachers in each class are relatively fixed in China. In this study, the teachers from different classes had different educational backgrounds, teaching experiences, and management modes, which may also have led to the clustering of depression and DSH at the class level. Moreover, social contagion plays an important role in young people's motivations for first-time DSH. If more friends engage in DSH, the adolescents are more likely to start DSH; the DSH risk among these adolescents is more than three times that of others. Some scholars provide a theory including imitation and hint to explain this phenomenon (56). However, the specific reasons for the clustering of depression and DSH in classes, as well as the forms and mediation of the interaction between classmates, warrant future investigation.

### Strengths and Limitations

This study has the following advantages. First, the longitudinal design and the bivariate multilevel logistic regression model not only enhanced the robustness of the interpretation of the results but also revealed whether the data had clustered at the high level. Second, exploring the common factors affecting both depression and DSH, which has been rare in past studies, could help maximize the benefits of an intervention. In other words, reducing the prevalence of depression also reduces the risk of DSH and vice versa. From the point of view of adolescents, intervention related to the common influencing factors of both can enable individuals to have healthier emotions and behaviors with minimal costs, and compliance may therefore be greater. Third, the findings showed that depression and DSH were clustered at the class level. To the best of our knowledge, this result has not been seen in past studies. It is feasible to identify and intervene with all students in classes with high rates of depression and DSH. This study also has several limitations. First, due to the high mobility of rural students, missing samples in the longitudinal studies were inevitable. Although the analysis showed no difference between the characteristics of the missing samples and the characteristics of the samples included in this study, some bias may exist. Second, the questionnaire had many subjective items, and self-report bias was difficult to avoid. Third, some important influencing factors for depression and DSH, such as academic performance and relationships with siblings, could not be included in this study, but should be explored in future studies. Fourth, This study used only one item to estimate DSH. Although we tested retest reliability of the DSH item by repeating measurements at 1-week intervals during the preliminary survey and the test retest reliability was excellent for this short term, the coefficient of long-term correlation was small for the 12-month period but had statistical significance. However, the measurement results with long interval time might also reflect the dynamic characteristics of DSH. Therefore, in future studies, it is best to establish test retest reliability of DSH measures for different assessment intervals and measures.

## Conclusion

Depression and DSH are concomitant and have a high prevalence rate in adolescents in western rural areas of China. There are common and unique influencing factors for depression and DSH. To maximize the benefits of intervention, common influencing factors should be given priority. In addition, close observation of unhealthy adolescents in some specific classes with higher rates of depression and DSH is needed.

## Data Availability Statement

The raw data supporting the conclusions of this article will be made available by the authors, without undue reservation.

## Ethics Statement

The studies involving human participants were reviewed and approved by Medical Ethical Committee of Sichuan University (No. 20140307). Written informed consent to participate in this study was provided by the participants' legal guardian/next of kin.

## Author Contributions

QL conceived and designed the study. SL and CS wrote the manuscript. FY analyzed the data. SS, MY, CT, and QZ collected the data. All authors read and approved the final manuscript.

## Funding

This study was supported by grants from the National Natural Science Foundation of China (No: 81472994), Applied Psychology Research Center Foundation of Sichuan Province, China (No: CSXL-182004), and the Fundamental Research Fund for Chinese Central Universities (No: skqy201212 and 20826041C4234).

## Conflict of Interest

The authors declare that the research was conducted in the absence of any commercial or financial relationships that could be construed as a potential conflict of interest.

## Publisher's Note

All claims expressed in this article are solely those of the authors and do not necessarily represent those of their affiliated organizations, or those of the publisher, the editors and the reviewers. Any product that may be evaluated in this article, or claim that may be made by its manufacturer, is not guaranteed or endorsed by the publisher.
