# Supplementary material for: Depression and Deliberate Self-Harm Among Rural Adolescents of Sichuan Province in Western China: A 2-Year Longitudinal Study
Source: Front Psychiatry. 2021 Sep 13;12:605785. doi: 10.3389/fpsyt.2021.605785 (PMC8473622; doi:10.3389/fpsyt.2021.605785)
Supplement: Supplementary file 1 [file Data_Sheet_1.PDF]

Appendix Table1

Appendix Table1 Correlation analysis among the three surveys of DSH ( score 1,2,3 and 4 for the answer of DSH)

| | The second follow-up survey | The fourth follow-up survey |
|---|---|---|
| Baseline data | 0.197* | 0.219* |
| The second follow-up survey | 1 | 0.311* |

* Significance at the 0.05 level
